# Editorial: Advances in the contributions of mathematics in the field of education and psychology

**DOI:** 10.3389/fpsyg.2024.1379669

**Published:** 2024-02-29

**Authors:** Belen Garcia-Manrubia

**Affiliations:** Department of Didactics of Mathematical and Social Science, University of Murcia, Murcia, Spain

**Keywords:** educational psychology, affective domain, mathematics, mathematical performance, mathematics education

Over the past decades, researchers have been investigating mathematics education and exploring factors associated with achievement in mathematics. Although one might assume that mathematics education and its positive outcomes are primarily linked to cognitive factors, such as spatial ability, critical thinking skills, or executive functioning, it is not understood without considering affective factors and their influence (Higbee and Thomas, 1999; Gómez Chacón, 2000). Nowadays, mathematics teaching is perceived as a multifaceted process influenced by a wide variety of factors such as students' self-concept, attitudes toward mathematics, mathematics anxiety, perceptions of the usefulness of mathematics, or motivation (Suárez-Álvarez et al., 2014). These variables may be related to different aspects involved in the teaching-learning situation, such as the teacher, the school, the family, or the student (Algani and Eshan, 2019). Therefore, mathematics education requires the efforts of educators and researchers to obtain an in-depth understanding of this complex process to reduce or eliminate the impact of cognitive and non-cognitive barriers to learning mathematics. The articles presented in this Research Topic address these issues from diverse perspectives.

This Research Topic opens with a study conducted by Xu and Qi, focusing on the mathematical problem-solving ability of middle school students in the compulsory education stage and the factors influencing their performance. The authors analyzed students' problem-solving competence by examining the impact of factors related to the school environment, such as location (urban or rural) and the teaching method. Additionally, they explored student-related factors, including self-efficacy and mathematics anxiety. The Research Topic continues with the study by Wang et al., which examines the impact of high school students' mathematical attitude determinants (attitude, subjective norms, and perceived behavioral control) on intentions, behavioral engagement, and mathematical performance. This study uses and extends the Theory of Planned Behavior (TPB) framework by incorporating mathematical performance. Using a different approach, Ramos-Galarza et al. carried out an intervention involving a program of Brain Gym exercises designed to enhance the learning of these key mathematical concepts among high school students. These concepts included the definition of rational numbers, problem-solving abilities, mathematical order relationships, and equivalent fractions. The authors aimed to improve the learning process through systematic exercises that stimulated neural connections between cerebral hemispheres.

The discussion of the topic progresses with Ling and Mahmud's study focused on sentence-based mathematical problem-solving skills. They used a qualitative research approach to detect challenges faced by teachers and the approaches they employ in addressing these challenges when teaching sentence-based mathematical problem-solving skills to primary school students. Exploring another element and its influence on mathematical education, Li et al. focused on studying the effect of Chinese family income on the intergenerational transmission of education using data provided by the China Family Panel Studies (CFPS). The authors further extend their analysis by examining whether the university expansion policy could alleviate potential differences in access to education motivated by the level of family income. Continuing with the topic, Yu et al. presented a study aimed at investigating how to measure mathematical self-efficacy meaningfully. For this purpose, they employed a multi-trait and multi-method design that included three traits (number and algebra, graphics and geometry, and synthesis and practice) and three methods to discuss [general-math-task-referenced self-efficacy, unconventional-math-problem-referenced self-efficacy, and Motivated Strategies for Learning Questionnaire (MSLQ) self-efficacy]. To verify the validity of the design, the authors constructed a confirmatory factor analysis (CFA) model.

Exploring student-related factors in the mathematics teaching-learning process, Puusepp et al. examined the development of potential associations between the mindset of primary school students and the attentional neural processing shown by students when receiving feedback on their mathematical performance. This feedback, whether positive or negative, has an impact on students that may depend, among other factors, on their beliefs and mindsets. To gain a better understanding of these student mindsets, neuroscientific research is employed, allowing the analysis of neural processes associated with the perception and cognition of feedback, avoiding limitations found in other data collection tools. Addressing the factors related to the methodology used in the mathematics classroom, Hui and Mahmud carried out a systematic literature review based on the PRISMA methodology and focused on game-based learning in mathematics education. This survey covered the influence of this teaching methodology on both the cognitive and affective domains. The topic persists in examining methodologies in this case to enhance the mathematical reasoning of elementary school students. The study conducted by Mahmud and Mohd Drus aimed to explore different types of oral questions used with elementary school students and their potential advantages in helping students improve their mathematical skills and reasoning.

Continuing the search for factors related to schools, students, and their families that affect students' mathematic outcomes, Molina-Muñoz et al. carried out an analysis of the potential impact that students' psychological and emotional factors may have on their mathematical literacy. The authors analyzed data from the Spanish sample of the 2018 edition of the Programme for International Student Assessment (PISA) using multilevel regression models. This allowed them to assess which factors had a positive Impact on students' mathematical competence and which resulted in a negative impact. Hernández de la Hera et al. focused on studying the possible causes that lead to the rejection of mathematics by high school and university students and their demotivation. They analyzed the relationships between attitudes toward mathematics, attitudes toward statistics, mathematical anxiety, and student self-efficacy, employing an artificial neural network for the backpropagation algorithm capable of predicting academic performance. Furthermore, based on the results obtained, they analyzed the implications these would have on mathematical education. The research proposed by Chen and Moc continued the quest for factors influencing learning by focusing their analysis on motivation and perceived family involvement among high school students. The study aimed to examine how these two factors relate to students' ability to cope with difficulties and challenges in their daily school life, using structural equation modeling. The Research Topic concludes with an article by Yu, who presented a systematic literature review focused on identifying key aspects and knowledge related to the neuroscientific basis of mathematical cognitive impairment and anxiety and their influence on educational practices, highlighting the interaction between cognitive processes and educational outcomes. In addition, this proposal provides suggestions for new strategies to practically address students' mathematic anxiety and cognitive impairments.

The contributions in this Research Topic provide a better understanding of the teaching and learning process of mathematics at various educational levels and the numerous factors influencing this process, related to aspects such as methodology, family, or the emotional dimension of the student. Additionally, the topic addresses aspects such as the analysis of methodologies, the neuroscientific basis, or proposing a measurement tool. Nevertheless, new issues arise that need to be addressed. For instance, it would be interesting to expand previous research on different aspects of the affective domain, such as anxiety toward mathematics, not only in students but also in teachers who already teach mathematics. The teacher's role is crucial for understanding the teaching-learning process. This Research Topic highlights new insights and explores additional aspects of mathematical education. Additionally, it raises new questions to advance the ongoing efforts in understanding the teaching and learning of mathematics, which is crucial for present society and even more so for future society.

## Author contributions

BG-M: Writing – original draft, Writing – review & editing.
